# Effectiveness of the Diabetes Prevention Program for Obesity Treatment in Real World Clinical Practice in a Middle-Income Country in Latin America

**DOI:** 10.3390/nu11102324

**Published:** 2019-10-01

**Authors:** Brianda Armenta-Guirado, Teresita Martínez-Contreras, Maria C. Candia-Plata, Julián Esparza-Romero, Raúl Martínez-Mir, Michelle M. Haby, Mauro E. Valencia, Rolando G. Díaz-Zavala

**Affiliations:** 1 Department of Health Sciences, University of Sonora, Blvd. Bordo Nuevo S/N, Blvd. Antiguo Ejido Providencia, Cajeme, Sonora 85010, Mexico; brianda.armenta@unison.mx; 2 Department of Chemical and Biological Sciences, University of Sonora, Blvd. Luis Encinas y Rosales S/N. Hermosillo, Sonora 83000, Mexico; teresita.martinez@unison.mx (T.M.-C.); haby@unimelb.edu.au (M.M.H.); mauro@ciad.mx (M.E.V.); 3 Department of Medicine and Health Sciences, University of Sonora, Blvd. Luis Encinas y Rosales S/N. Hermosillo, Sonora 83000, Mexico; carmen.candia@unison.mx; 4 Diabetes Research Units, Department of Public Nutrition and Health, Research Center for Food and Development CIAD, A.C., Camino Gustavo Enrique Astiazarán Rosas No. 46 Col. La Victoria. Hermosillo, Sonora 83000, Mexico; julian@ciad.mx; 5 Department of Psychology and Communication, University of Sonora, Blvd. Luis Encinas y Rosales S/N. Hermosillo, Sonora 83000, Mexico; raulmmir@psicom.uson.mx

**Keywords:** obesity treatment, weight loss, lifestyle interventions, effectiveness, diabetes prevention, Mexico, Latin America

## Abstract

The Diabetes Prevention Program (DPP) is effective for the prevention of type 2 diabetes by weight loss with diet and physical activity. However, there is little evidence as to whether this program could be translated into real-world clinical practice in Latin American countries. The objective of this work was to evaluate the effectiveness of the DPP for the management of overweightness and obesity at 6 and 12 months in clinical practice in Mexico. This was a non-controlled intervention study implemented in five public clinics in northern Mexico. Two hundred and thirty-seven adults aged 45.7 ± 9.9 years with a Body Mass Index (BMI) of 34.4 ± 5.4 kg/m^2^ received group sessions with an adaptation of the DPP, in addition to nutrition counseling. One hundred and thirty-three (56%) participants concluded the 6 month phase. They showed a significant weight loss, ranging from 2.76 ± 4.76 to 7.92 ± 6.85 kg (*p* ≤ 0.01) in the clinics. The intention-to-treat analysis showed a more conservative weight loss. Participant retention at the end of 12 months was low (40%). The implementation of the DPP in different public clinics in Mexico was effective in the management of obesity in the short term, but better strategies are required to improve participant retention in the long term.

## 1. Introduction

The number of adults with diabetes quadrupled globally from 1980 to 2014, affecting 422 million adults in 2014 [1]. Currently, this disease is considered one of the main causes of morbidity and mortality in the world [2].

A more rapid increase in the prevalence of diabetes has been documented in low- to middle-income developing countries [1,2,3]. Numerical estimates have been made, projecting that by the year 2025 the number of people with diabetes in developing countries, such as in Latin America, will increase by 170%, compared to an increase of 42% in developed countries [4]. Diabetes, in conjunction with other noncommunicable diseases, is one of the main causes of death in these countries [3]. Around 70% of the global prevalence of diabetes is found in this region [5].

Overweightness and obesity are the most important modifiable risk factors for the development of type 2 diabetes [6]. Epidemiological studies show that the prevalence of obesity increased twice as fast in Latin America when compared to developed countries [7,8]. In the last 30 years, this region has experienced important demographic and socioeconomic changes, considered to be crucial risk factors [9,10]; this lead to a “Nutritional Transition”, referring to dietary changes explained by an increase in access to food, combined with a decrease in physical activity [11]. Such is the case of Peru, a country with the highest density of fast food restaurants in the world, while Brazil has quintupled the consumption of sweets and junk food in the last three decades [12]. Chile, on the other hand, consumes more than half of its food in processed goods [12]. Argentina has one of the lowest physical activity rates in the world [7], and Mexico is one of the largest consumers of sugar-sweetened beverages worldwide [13].

In Mexico in particular, diabetes is more common and has a greater effect on mortality than in developed countries, causing at least one third of deaths between 35 and 74 years of age [14]. This medium-income country has the highest documented increase in obesity prevalence worldwide (2% per year), together with a prevalence of diabetes of 14.4% [15] with insufficient glycemic control associated with a much worse prognosis than that observed in high-income countries [14]. 

While the impact of diabetes on our region continues to increase, the efforts in preventive research and its translation to the community have been limited. There are programs that have been successful in preventing type 2 diabetes through lifestyle changes, such as the one implemented in the U.S. Diabetes Prevention Program (DPP). This study demonstrated that an intensive lifestyle intervention program—low-fat diet, physical activity, behavior change strategies, frequent visits in the initial phase, and trained personnel—effectively promotes moderate weight loss (7 kg) at 1 year of treatment and a reduction in the incidence of diabetes by 58% at 2.8 years [16]. The DPP adapted protocol has shown positive results in weight loss in individuals with obesity and diabetes with multiple benefits for health [17]. On the basis of the efficacy of the Diabetes Prevention Program, this program has been translated and evaluated in real world scenarios, including primary care clinics, churches, community centers, and others, with promising results (2.7% to 6% of weight loss from 3 months to 2 years and improvements in risk factors) [18]. Considering the evidence of effectiveness of the DPP shown in both randomized controlled trials and translational trials, the U.S. Health authorities, with the support of Congress, established the National Diabetes Prevention Program in 2010. The Program included, among other measures, making a low-cost adaptation of the DPP protocol available to at-risk populations in order to reduce their incidence of type 2 diabetes [19], and a recent evaluation of this program showed positive results [20]. To date, most translational studies have been conducted in developed countries, such as the U.S. and Europe, with little information on the effectiveness of translating the DPP in Mexico and other Latin American countries [21].

## 2. Materials and Methods 

### 2.1. Study Design

The primary objective of the present study was to evaluate the effectiveness of the translation of the DPP for the management of overweight and obesity (weight loss) at 6 and 12 months in clinical practice in five different points of health care in Mexico, a middle-income country in Latin America. Secondarily, the effect of the program on other obesity parameters Body Mass Index (BMI), waist circumference, and body fat percentage), percentage of weight loss goals, physical (systolic and diastolic blood pressure), biochemical parameters, and aspects of mental health (perceived stress scale, symptoms of depression, and health-related quality of life) were estimated.

The present study was a non-controlled intervention clinical study of effectiveness with a translational approach of 6 and 12 months of follow-up with a post-test pre-test design implemented in five public clinics from northern Mexico. The methods have been described in detail in the protocol of this study [22]. The protocol was approved by the Research Bioethics Committee of the Department of Medicine and Health Sciences of the University of Sonora (10 April 2015) and by the Research Committee of the Medical Center “Dr. Ignacio Chávez” (CEI-015-2015). All participants signed an informed consent before beginning the intervention. The intervention program did not present any cost for the participants, nor did they obtain any economic remuneration for participating. The study consisted of two phases: (1) training and standardization of the health providers of the participating clinics in August 2015 (an additional group of nutrition interns who replaced the first group received the training later, given that the internship duration is 1 year) and (2) implementation of the program and its evaluation at 6 and 12 months from September 2015 to April 2017.

### 2.2. Participating Clinics

Five clinics from Hermosillo, Sonora, Mexico were included. Clinic 1 is a public university clinic that regularly provides the Mexican adaptation of the DPP at low cost, as well as health promotion programs to the community. Clinics 2 and 3 are within public hospitals. Clinic 2 is part of a public hospital of second level specialties with third level care procedures. It provides medical services to the general population of the state of Sonora at low cost. Clinic 3 is part of a public hospital that includes different medical specialties and serves employees of agencies affiliated with the government of the state of Sonora. Clinics 4 and 5 are primary care clinics. Both are urban public clinics that provide primary care services.

### 2.3. Training for Health Providers

Health providers from each clinic were trained, including nutrition interns, certified nutritionists, and primary care physicians who worked or provided their service in the clinic on a regular basis. They attended a course focused on the clinical evaluation and management of patients with obesity, anthropometric measurements standardization, and blood pressure, as well as the implementation of an adaptation of the DPP protocol: Lifestyle Balance [22], available at http://www.diabetesprevention.pitt.edu/index.php/2011-dpp-group-lifestyle-balance-curriculum-spanish/. The Program was adapted for the Mexican population by the authors, and consisted of 32 topics, organized in 25 sessions, which address aspects of nutrition, physical activity, and a behavior change protocol, considering cultural adjustments for the Mexican context [22].

### 2.4. Recruitment

Subjects were invited to participate through social networks, such as Facebook advertising, posters, and printed flyers in participant clinics. Nutrition interns in each clinic promoted the value of the program for achieving weight loss and reducing obesity related diseases to potential subjects while they waited for their consultation with the doctor. Additionally, the doctors and nurses from each clinic actively referred patients to the study. Candidates interested in participating in the study had an appointment to confirm that they met the inclusion and exclusion criteria. Once patients were recruited in each clinic, they were divided into groups of 25–50 people for group sessions. 

### 2.5. Inclusion Criteria

The nutrition interns at the different participating clinics were in charge of recruiting participants for the study, considering the following inclusion criteria: adults (age ≥18 and ≤65 years), suffering from being overweight or obesity (BMI ≥25 kg/m^2^ and ≤50 kg/m^2^) with availability and motivation to attend the intervention program, to attended at least one individual consultation and one group session, in addition to signing and accepting the informed consent. Since the study had a translational approach, all patients who could benefit from the program were included, even if they presented conditions that could interfere with their body weight (i.e., depression) or were taking medications with effects on weight (sulfonylureas, metformin, etc.). People who could not read were included if another person was willing to accompany them to the sessions and explain the content. The exclusion criteria were women who were pregnant or breastfeeding within the last 6 months, people with bariatric surgery, a history of glycated hemoglobin A1c ≥9%, patients with insulin treatment, systolic blood pressure ≥160 mm/Hg, and those who could be negatively affected by weight loss or physical activity.

### 2.6. Study Intervention

The duration of the intervention was 1 year. The first 3.5 months were intensive, with a weekly group session (14 sessions) that included material from the Lifestyle Balance behavior change protocol and between 1 and 4 individual consultations for nutritional advice per month, in accordance with the agreement between the health provider and the patient, considering time availability in the clinic. Patients attended group sessions and individual consultations at different times (usually on different days). Intensity was lower from 3.5 to 6 months, with a biweekly visit to the group sessions and an individual consultation per month. The participants attended a group session and an individual consultation per month in the 6 to 12 months. The conditions at each of the participating clinics to carry out the study were typical—they did not change their usual care program (for example, the primary care clinics and public hospitals operated in the morning and the university clinic in the morning and afternoon), and health care providers were not asked to see patients outside their normal work schedules to implement the study. The nutrition interns, who lacked previous experience in obesity management, recruited the patients and implemented the intervention. In addition to the study intervention, participants could receive the conventional medical care.

### 2.7. Behavior Change Protocol

Participants received a printed manual of the Mexican adaptation of the Lifestyle Balance program [22]. This manual includes topics of nutrition and physical activity, as well as behavior change strategies, such as self-monitoring, stimulus control, and positive reinforcement, among others. The manual includes three physical activity sessions (combining theory and practice) in which the following topics are explained: the different types of physical activity, recommendations about how to increase the time of activity, the use of accelerometers, how to perform exercise safely, how to find time to exercise, how to do exercise routines at home, and how to increase intensity, among others. For the Mexican adaptation of the Lifestyle Balance program, cultural adaptations were considered, and we added topics such as “Food weighing”, “Food groups”, “Portions sizes”, “How to design your own menu”, “Diabetes prevention”, etc. (Table 1). In addition, the research group developed activities that could be implemented in each of the group sessions. The original “Group Lifestyle Balance” Patient manual and the provider manual are available for free at http://www.diabetesprevention.pitt.edu/index.php/2011-dpp-group-lifestyle-balance-curriculum-spanish/. 

The goal for each participant was to lose 10% of their initial body weight and, to achieve it, they had to gradually reach 150 min of physical activity per week, as well as reducing fat intake in their diet.

### 2.8. Individual Consultations for Nutrition Counseling

The first individual consultation lasted between 40 to 60 min and subsequent consultations were 20 to 30 min. Each participant completed a nutritional assessment (anthropometric, biochemical, dietetic, and clinical evaluation). The total energy expenditure of each participant was estimated by calculating their resting energy expenditure (using predictive equations) and multiplying by a physical activity factor. This value was considered when prescribing a hypocaloric diet in the range of 1200–1800 kcal. The use of a meal replacement was recommended to improve weight loss; participants were able to buy commercial meal replacements or make a milkshake at home with foods prescribed by the nutrition intern. They also had the option of choosing a meal plan prepared by a nutritionist if they did not want to take meal replacements. In these appointments, the nutritionist reviewed the progress of the participants with their goals and helped them to solve problems related to adherence to the diet and physical activity. 

### 2.9. Study Measures

Measurements of outcome variables were taken at the beginning of the study and at 6 and 12 months by the research staff at the University of Sonora [22]. The measurement techniques described below are appropriately referenced in the study protocol [22]. Body weight and height were measured following standard techniques, in a SECA mBCA (medical body composition analyzer, SECA Gmbh & Co. Kg, Hammer Steindamm 9-25, Hambur, Germany) and SECA stadiometer, model 284 (Seca Gmbh & Co. Hammer Steindamm 9-25, Hambur, Germany; capacity 30–220 cm) respectively. Waist circumference was measured at umbilical level with a fiberglass anthropometric tape (GÜLICK brand, Leverkusen, Germany, 0–150 cm). Body fat percentage was estimated by electrical bioimpedance with the same SECA mBCA equipment. Systolic and diastolic blood pressure were measured in duplicate with a digital baumanometer, following established guidelines with an Omrom equipment, (model HEM-907XL, Omrom, Osaka, Japan). Validated questionnaires were used to evaluate mental health aspects, such as depression (Beck Depression Inventory), health-related quality of life (SF-36 survey), and stress (Perceived Stress Scale PSS-14) (the impact of the program on these variables will be reported in a separate publication). Biochemical parameters were determined by colorimetric techniques (RANDOX, Crumlin, U.K.) in serum from fasting venous blood samples, including glucose, total cholesterol, low-density lipoprotein-cholesterol (LDL-cholesterol), high-density lipoprotein-cholesterol (HDL-cholesterol), triglycerides, and the hepatic enzymes aspartate aminotransferase and alanine aminotransferase. In addition, measured fasting insulin and fasting glucose were used to calculate the HOMA-IR (homeostatic model assessment for insulin resistance). Biochemical analyses were carried out at the Clinical Biochemistry Laboratory, and all other measurements at the Nutritional Health Promotion Center, both from the University of Sonora Campus Hermosillo. 

### 2.10. Statistical Analysis

A sample size of 14 participants per clinic was calculated for the main study variable (change in body weight), using a mean weight loss of 4.2 kg and a standard deviation of 5.6 kg from a previous study with one-year duration [23,24]. A two-tailed paired *t*-test with an α = 0.05 and a power of 80% was used. However, we aimed to recruit 50 participants per clinic to allow for attrition and in consideration of the study’s translational purposes. Data were presented as means and standard deviation (mean ± SD) and proportions. A paired *t*-test or a Wilcoxon signed-rank test (for variables with non-normal distribution) was used to evaluate change from baseline to follow-up for the main variable and the secondary variables for each center. The main outcome variable and the secondary variables at 6 and 12 month follow-up were analyzed in the participants who completed each phase. Additionally, we also used a modified intention-to-treat (ITT) analysis, given that this was not a randomized controlled trial but included all participants in the study regardless of subsequent withdrawal from treatment or deviation from the protocol. An effort was made to obtain data from the participants who left the study at 6 and 12 months, and these were included in the intention-to-treat analysis. For the subjects who did not attend the 6 and 12 month measurements, the final value was substituted for the baseline value (baseline-observation-carried forward) for this conservative analysis [25].

In addition, the differences in primary and secondary variables between clinics at 6 and 12 months were evaluated using the one-way ANOVA or Kruskal–Wallis test (with Bonferroni or Dunn’s post hoc analysis) for continuous variables with normal or non-normal distribution, respectively, and chi-square analysis (χ^2^) for categorical variables. The two-sided level of significance was set at α ≤ 0.05 as a criterion of statistical significance. The analyses were carried out with the NCSS statistical software version 10 (Number Cruncher Statistical System for Windows, Kaysville, UT, USA) and Stata Statistical Software (Version 14. StataCorp LP, College Station, TX, USA). The review of the study database and the statistical analyses were done by the research team. Nevertheless, these were also corroborated by personnel external to the study, from the Research and Statistical Consulting Laboratory of the Mathematics Department of the University of Sonora.

## 3. Results

### 3.1. Participants, Baseline Characteristics, and Attendance at Scheduled Visits

**Figure 1:**
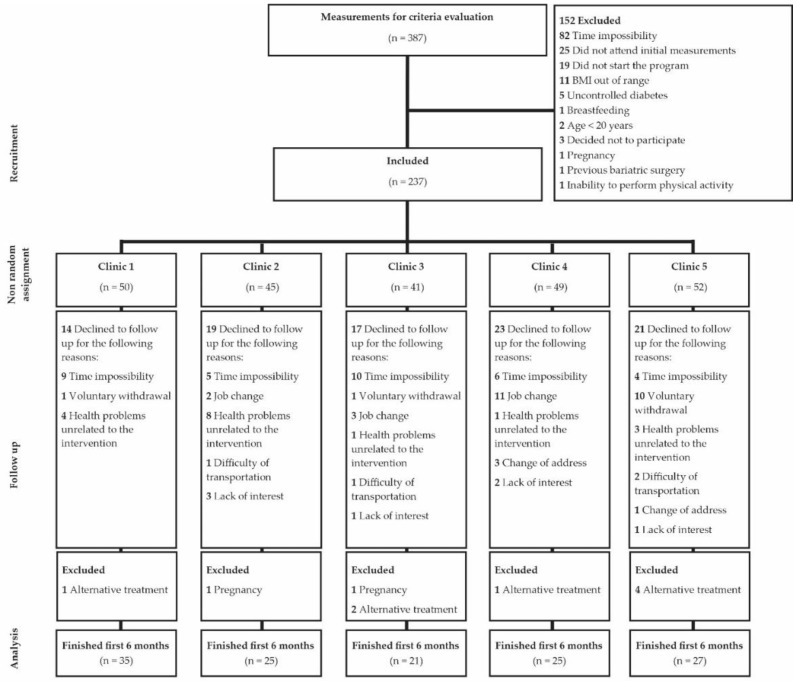
Flow diagram of study participants in the five clinics through the 6 months of intervention.

Three hundred and eighty-seven individuals from the five clinics attended the invitation to participate in the study from September 2015 to April 2016. Attempts were made to enroll 250 participants (50 per clinic). As some participants did not show up for the intervention and some were excluded, the final sample was 237. It was not possible to add more individuals because the intervention had established dates for group sessions. Among the top reasons for non-participation were schedule incompatibility, did not complete the baseline measurements, or had a BMI out of range (Figure 1).

There was a high drop-out of the study participants at 12 months (60%), so the present study focused mainly on the results at 6 months. The 12 month results are briefly described at the end of this section. However, these should be considered with caution because of the potential biases involved in an analysis with such low retention. 

More than half of the participants included in the study (133/237 or 56.1%) completed the first six months of intervention, with a slight variation in retention between different clinics (Figure 1). Eighty percent of the participants who completed the 6 month measurements were female, had an average age of 46 years, and had grade I obesity (BMI 34.4 ± 5.39 kg/m^2^). The participants reported a previous diagnosis of hypertension (24%), type 2 diabetes (15%), and/or hypothyroidism under treatment (13%). There were no participants with pathologies that significantly affected body weight (e.g., Cushing’s syndrome, hypothalamic obesity, etc.). Table 2 shows the baseline characteristics of the participants who completed the study in each clinic. No differences were observed in most baseline characteristics between those who abandoned the study and those who completed the 6 month measurements *(p* > 0.05) (Supplementary Materials, Table S1). However, body weight (*p* < 0.02) was higher in dropouts than in completers in clinic 5, and some other secondary outcome variables were different between clinics.

After six months of intervention, participants in Clinic 1 had attended 11.8 ± 4.9 group sessions of the 19 planned in this period. In the rest of the clinics, attendance was 12.2 ± 6.1 for clinic 2, 14.8 ± 5.4 for clinic 3, 10.8 ± 5.0 for clinic 4, and 13.2 ± 5.8 for clinic 5. Individual nutrition consultations recorded an average attendance of 16.0 ± 6.7 in clinic 1, 8.3 ± 4.3 in clinic 2, 10.1 ± 3.6 in clinic 3, 12.6 ± 8.5 in clinic 4, and 8.3 ± 4.8 in clinic 5. It should be mentioned that the number of individual consultations offered by the clinic could vary accordingly to the protocol from a minimum of 6 to a maximum of 24, depending on the agreement between the provider and participant, as well as time availability in the clinic.

### 3.2. Change in Primary Outcome at 6 Months

**Figure 2:**
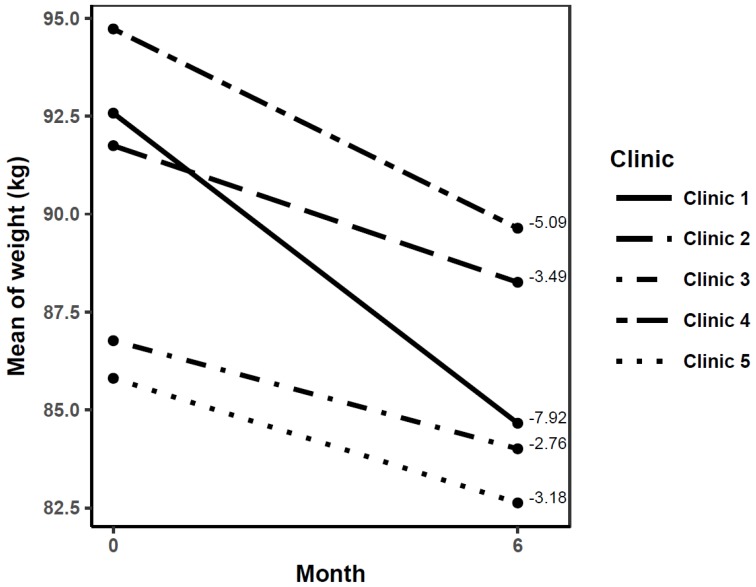
Change in body weight of participants who completed the 6-month phase of lifestyle intervention per clinic. Change in body weight (mean ± SD, 95% CI) in each clinic: 1 (−7.92 ± 6.84 kg, 95% CI −10.3, −5.57), 2 (−3.49 ± 4.12 kg, 95% CI −5.20, −1.79), 3 (−2.76 ± 4.76 kg, 95% CI −4.92, −0.60), 4 (−5.09 ± 5.03 kg, 95% CI −7.16, −3.00), and 5 (−3.18 ± 3.91 kg, 95% CI −4.73, −1.63).

At the beginning of the intervention, participants who completed the first 6 months of intervention had a body weight that varied from 85.8 ± 13.0 to 94.7 ± 21.2 kg among the five clinics (Table 2). The participants at the five clinics showed a significant effect on the primary variable of the study (weight loss) at 6 months post-intervention (*p* < 0.0001 for clinics 1 and 4; *p* < 0.001 for clinics 2 and 5; clinic 3, *p* < 0.05). The amount of weight loss varied between the clinics, with the greatest effect in clinic 1 (7.92 ± 6.85 kg, *p* < 0.0001) and lowest in clinic 3 (2.76 ± 4.76 kg, *p* < 0.05) (Figure 2). A significant difference (*p* < 0.001) in weight loss between clinics 1 vs. 2, 3, and 5 was observed. A 5% weight loss at 6 months was achieved by 62.8% of the participants in clinic 1, while in clinics 2, 3, 4, and 5 it was achieved by 48%, 28.5%, 48%, and 33.3%, respectively. 

Furthermore, we observed a positive effect on body weight reduction with the intention-to-treat analysis (*p* ≤ 0.05 for all clinics); however, as expected, the reduction was more moderate. Weight loss was 5.96 ± 6.55 kg in clinic 1, 2.11 ± 3.55 kg in clinic 2, 1.75 ± 4.30 kg in clinic 3, 2.32 ± 4.64 kg in clinic 4, and 1.61 ± 3.28 kg in clinic 5; significant differences were detected (*p* < 0.0001) between clinic 1 and clinics 2, 3, 4, and 5.

#### Changes in Secondary Variables at 6 Months 

In addition to the effect on body weight, we observed significant improvements in other obesity parameters, such as BMI, waist circumference, body fat percentage, as well as blood pressure (Table 3). In the intention-to-treat analysis, significant but more moderate effects were observed in the decrease in BMI (*p* < 0.05), waist circumference (*p <* 0.05), and body fat percentage (*p* < 0.05) in all clinics, while only 3 and 2 out of 5 clinics were significant (*p* < 0.05) for systolic and diastolic blood pressure, respectively.

### 3.3. Follow-Up of Participants at 12 Months 

There was a very high drop out of study participants at 12 months. Only 40.0% (*n* = 95) of the participants who started the study were evaluated, varying between clinic 1 (44.0%), clinic 2 (33.3%), clinic 3 (41.4%), clinic 4 (38.7%), and clinic 5 (42.3%). Reasons why participants reported leaving the study at this stage and results for other outcome variables are shown in the Supplementary Materials.

## 4. Discussion

The implementation of the DPP adapted for obesity treatment in five different points of health care in Mexico, a middle-income country in Latin America, is effective when applied by staff who typically provide care to patients in real-world clinical practice, at least in the short term. This is one of the first studies of its type in Mexico and Latin America [21], where an accelerated increase in the prevalence of type 2 diabetes is expected [7]. However, it should be noted that there was a moderate retention of participants at 6 months and a very low retention at 12 months, which suggests that, although the results are promising, strategies to retain patients need to be improved.

Effectiveness of the program was evaluated by weight loss in five clinics. Body weight reduction after six months of intervention in participants who completed this phase was significant in all clinics, ranging from 2.76 kg to 7.91 kg (3.2% to 8.6% of baseline body weight, whereas in intention-to-treat analysis results were more conservative, ranging from 1.61 kg to 5.96 kg (1.7% to 6.4% of baseline body weight). These results are similar to those in other translational studies with the Diabetes Prevention Program in other countries where weight reduction of 1 kg to 7.27 kg was observed at 6 months [24,28,29,30]. A systematic review of translational studies of diabetes prevention programs over the past 15 years showed that participants achieve a 12 month weight loss between 0.45 to 7.70 kg [31]. To our knowledge, there is only one other translational study published with the Diabetes Prevention Program in Latin America [32,33], which was also conducted by us and achieved a weight loss of 4.70 kg at three months [33]. Furthermore, the results of the current study (2 to 6 kg at 6 months by intention-to-treat analysis) are superior to those regularly obtained with traditional treatment in clinical practice—with monthly or less frequent consultations—where weight gain has been observed [34] or with minimal weight loss (usually less than 1 kg at 12 months) [34,35].

A strength of the current study is that it reflects conventional clinical practice, with broader inclusion criteria, including patients with multiple diseases, such as type 2 diabetes, depression, hypothyroidism, and use of weight-increasing drugs, among others, who are often excluded from efficacy studies [36]. The need to have results in “typical” patients has been highlighted rather than in those who, because they fulfil numerous criteria, sometimes do not resemble patients present in usual clinical practice. Furthermore, we should mention that these results were obtained by health professionals who care for this population on a regular basis, and not by a staff of expert researchers. This type of phase 2 translational research study is an essential complement to controlled efficacy clinical trials (translational research phase 1) [37] in order to have a more complete picture for decision making.

We were very careful in ensuring that the conditions for implementation were the same as in usual clinical practice, without modifying clinic hours, nor did we use any economic or other types of incentives to improve adherence to and completion of the intervention [38] or to encourage attendance at final measurements [28,38]. There was also no additional support for gym memberships to perform physical activity, for delivery of metering devices, or for other factors [39]. 

Nutrition interns without any previous experience implemented the program, increasing its potential for generalization. This is consistent with previous observations where even lay educators can obtain good reductions in body weight using the DPP in translation studies [40]. Likewise, this model with interns represents an economically viable strategy for countries with limited resources, such as Mexico and many countries in Latin America that do not have the capacity to hire additional personnel to implement the program. In Mexico, nutrition interns complete a 12 month placement as part of their social service and are paid a very small salary (5% of the normal salary). The Diabetes Prevention protocol has demonstrated its cost-effectiveness in people at high risk of developing diabetes [41]. Moreover, efforts have been made to achieve greater access to the population by adapting this protocol through civil associations with wide coverage such as the Young Men’s Christian Association (YMCA) in the United States. The implementation of the DPP protocol in the YMCA partners has shown positive and cost-effective results [30,42] but is not feasible in Mexico at this time. 

Within the limitations of the study was the moderate retention of participants (56%) at 6 months, which seems to be the reality of 6 month translational studies (57%–92%) [24,28,30,39]. The results at 12 months, as we mentioned previously, should be taken with caution because the effect has probably been overestimated because of high dropout rates. The pre-posttest design has clear limitations to infer causality and it is certainly better to have a randomized controlled trial design. Despite this, the objective of this work was to transfer this validated program to the community. Additionally, it has been observed that participants of the control groups in weight loss studies have minimal weight loss at 12 months (-0.8kg (95% CI: −1.1 to −0.4)) [43].

## 5. Conclusions

This study showed that the implementation of the DPP adapted for obesity treatment was effective in five different sites of health care attention when applied by personnel who typically provide care to patients in real-world clinical practice in Mexico, a middle-income country in Latin America. These findings, together with other DPP translation studies, show that this program has a high potential to be used in obesity treatment and to reduce the incidence of type 2 diabetes. 

## Figures and Tables

**Table 1:** Adaptation of the Diabetes Prevention Program (DPP) protocol “Group Lifestyle Balance Program”.

Hours	Topics
**3.5 months (weekly)**	Session 1. Welcome to the Lifestyle Balance Program^®^ Session 2. Be a fat and calorie detective Session 2.1 Reading a nutrition label Session 2.2 Cooking demonstration and food weighing * Session 3. Move those muscles Session 4. Food groups and portion sizes * Session 5. Healthy eating and calorie balance tilting Session 6. Take control of what’s around you Session 7. How to design your own menu (Mexican System for Food Equivalents) * Session 8. Problem solving Session 9. Four key points to eating out healthily and the slippery slope of lifestyle change Session 10. Make social cues work for you and activity plan kickoff Session 11. You can manage stress Session 12. How to feel motivated
**3.5–6 months** **(biweekly)**	Session 13. Obesity risks * Session 14. Diabetes prevention * Session 15.1 Heart health and cholesterol * Session 15.2 Heart health and hypertension * Session 16. Relationship between obesity and cancer *
**6–12 months** **(monthly)**	Session 17. Getting ready for long-term self-control and adjust your thoughts for long-term self-control Session 18. More volume, less calories and conscious eating Session 19. Strengthen your exercise program Session 20. Stretching: the truth about flexibility Session 21. Rise for your health Session 22. Looking at the past and looking at the future

* Additional session to the original program.

**Table 2:** Baseline characteristics of participants completing the 6 months of intervention (*n* = 133).

Variable	Clinic 1 *n* = 35	Clinic 2 *n* = 25	Clinic 3 *n* = 21	Clinic 4 *n* = 25	Clinic 5 *n* = 27
Female, *n* (%)	26 (74.2)	18 (72.0)	18 (85.7)	20 (80.0)	24 (88.9)
Age, mean ± SD, year	43.5 ± 11.7	44.8 ± 9.84	48.1 ± 8.05	47.2 ± 8.10	46.4 ± 9.94
Education, *n* (%)					
Elementary school	2 (5.71)	10 (40.0)	4 (19.1)	5 (20.0)	3 (11.1)
High school	15 (42.9)	7 (28.0)	7 (33.3)	8 (32.0)	7 (25.9)
College/University	14 (40.0)	6 (24.0)	9 (42.9)	9 (36.0)	15 (55.6)
Postgraduate	4 (11.4)	2 (8.00)	1 (4.76)	3 (12.0)	2 (7.41)
Monthly income, *n* (%) ^a^					
<U.S. $296	11 (31.4)	8 (32.0)	1 (4.76)	7 (28.0)	3 (11.1)
U.S. $296 to $592	4 (11.4)	6 (24.0)	6 (28.6)	9 (36.0)	11 (40.7)
U.S. $592 to $1,185	11 (31.4)	4 (16.0)	12 (57.1)	7 (28.0)	7 (25.9)
U.S. $1,185 to $1,777	6 (17.1)	4 (16.0)	1 (4.76)	0 (0.00)	1 (3.70)
≥U.S. $1,777	3 (8.57)	3 (12.0)	1 (4.76)	2 (8.00)	5 (18.5)
Marital status, *n* (%)					
Single	10 (28.6)	4 (16.0)	2 (9.52)	5 (20.0)	9 (33.3)
Married	21 (60.0)	19 (76.0)	17 (81.0)	16 (64.0)	16 (59.3)
Divorced	3 (8.57)	2 (8.00)	2 (9.52)	4 (16.0)	0 (0.00)
Widowed	1 (2.86)	0 (0.00)	0 (0.00)	0 (0.00)	2 (7.41)
Diseases by self-report, *n* (%)					
Type 2 diabetes	2 (5.71)	5 (20.0)	5 (23.8)	4 (16.0)	4 (14.8)
Hypertension	6 (17.1)	6 (24.0)	8 (38.1)	6 (24.0)	6 (22.2)
Abnormal lipids	2 (5.71)	3 (12.0)	0 (0.00)	2 (8.00)	1 (3.70)
Hypothyroidism	4 (11.4) ^3^	1 (4.00) ^3^	7 (33.3) ^1,2,4^	1 (4.00) ^3^	5 (18.5)
Depression	1 (2.86)	2 (8.00)	2 (9.52)	1 (4.00)	0 (0.00)
Hypoglycemic drugs, *n* (%) ^b^	1 (2.86)	5 (20.0)	5 (23.8)	4 (16.0)	4 (14.8)
Height, mean ± SD, m	1.63 ± 0.08	1.63 ± 0.07	1.60 ± 0.07	1.63 ± 0.07	1.61 ± 0.07
Weight, mean ± SD, kg	92.6 ± 19.6	91.7 ± 11.8	86.8 ± 13.2	94.7 ± 21.2	85.8 ± 13.0
Body mass index, mean ± SD, kg/m^2^	34.7 ± 5.20	34.6 ± 3.98	34.2 ± 6.11	35.4 ± 6.54	33.2 ± 5.17
Waist circumference, mean ± SD, cm	107 ± 13.0	109 ± 10.6	105 ± 12.4	110 ± 16.5	103 ± 10.5
Body fat percentage, mean ± SD ^c^	44.6 ± 5.42	44.7 ± 5.78	45.2 ± 5.57	44.8 ± 5.32	45.3 ± 6.24
Systolic blood pressure, mean ± SD, mmHg	122 ± 12.3	119 ± 11.8	130 ± 18.3	125 ± 16.6	119 ± 11.1
Diastolic blood pressure, mean ± SD, mmHg	77.3 ± 6.81	74.8 ± 9.98	78.0 ± 12.2	75.8 ± 11.7	75.7 ± 7.75
Fasting glucose, mean ± SD, mg/dL	86.3 ± 32.2	97.9 ± 42.6	86.4 ± 15.4	91.2 ± 24.9	84.7 ± 26.2
Fasting insulin, median (P25, P75), µU/mL ^c^	6.63 (5.11, 11.8)	6.06 (4.39, 11.9)	6.07 (4.49, 8.60)	5.27 (3.24, 8.17)	4.93 (4.06, 8.46)
HOMA-IR, median (P25, P75) ^d^	1.31 (1.01, 2.39)	1.21 (0.81, 2.97)	1.15 (0.86, 2.37)	1.06 (0.60, 1.86)	0.95 (0.71, 1.91)
Triglycerides, mean ± SD, mg/dL	138 ± 81.9	161 ± 71.6	177 ± 156	164 ± 68.4	135 ± 74.6
Total cholesterol, mean ± SD, mg/dL	169 ± 27.5 ^4^	194 ± 50.2	181 ± 57.4	197 ± 34.0 ^1^	190 ± 46.9
High-density lipoprotein-cholesterol (HDL-cholesterol), mean ± SD, mg/dL	53.0 ± 10.5 ^3^	50.6 ± 17.4	42.2 ± 12.9 ^1^	45.9 ± 11.3	43.6 ± 11.8
Low-density lipoprotein-cholesterol (LDL-cholesterol), mean ± SD, mg/dL	89.3 ± 28.1 ^4,5^	111 ± 44.3	103 ± 36.5	119 ± 31.5 ^1^	119 ± 45.7 ^1^
Aspartate aminotransferase, mean ± SD, U/L	14.9 ± 5.78	16.3 ± 3.39	18.9 ± 6.98	18.2 ± 8.57	20.6 ± 9.99 ^d^
Alanine aminotransferase, mean ± SD, U/L	14.7 ± 4.20 ^5^	17.1 ± 4.54	15.6 ± 4.64	17.8 ± 6.54	19.9 ± 7.58 ^1^
Metabolic syndrome, *n* (%) ^e^	12 (34.3)	15 (60.0)	13 (61.9)	15 (60.0)	10 (37.0)
Metabolically healthy, *n* (%) ^f^	23 (65.8)	10 (40.0)	8 (38.1)	10 (40.0)	16 (59.3)
Metabolically unhealthy, *n* (%) ^g^	12 (34.3)	15 (60.0)	13 (61.9)	15 (60.0)	11 (40.7)

^1^ Clinic 1, ^2^ clinic 2, ^3^ clinic 3, ^4^ clinic 4 and ^5^ clinic 5. *p*-value: one-way ANOVA or Kruskal–Wallis test (with Bonferroni or Dunn’s post hoc analysis) for continuous variables with normal or non-normal distribution, respectively, and chi-square analysis (χ2) for categorical variables. The superscript numbers indicate the clinics in which there were significant differences, *p* < 0.05 by the Bonferroni test. ^a^ Exchange rate: 16.88 Mexican pesos per U.S. dollar as of September, 2015. ^b^ Hypoglycemic drugs used in patients with diabetes: metformin (*n* = 19) and sulfonylureas + metformin (*n* = 3). ^c^ Percentage of fat and fasting insulin (*n* = 131). ^d^ HOMA-IR (homeostatic model assessment for insulin resistance) *n* = 129. Conventional unit conversion factors: to convert mg/dL glucose to mmol/L, multiply mg/dL by 0.0555; to convert mg/dL triglyceride to mmol/L, multiply mg/dL by 0.0113. To convert mg/dL total cholesterol, LDL-C, and HDL-C to mmol/L, multiply mg/dL by 0.026. ^e^ Metabolic syndrome: definition according to the National Expert Panel on Detection, Evaluation, and Treatment of High Blood Cholesterol in Adults (NCEP-ATP-III) update from 2005. Three or more of the following risk factors—blood pressure (systolic/diastolic ≥130/85 mm Hg), triglycerides (≥150 mg/dL), HDL-cholesterol (<40 mg/dL in men and <50 mg/dL in women), fasting glucose (≥100 mg/dL), or taking medicine for the mentioned risk factors, abdominal obesity (waist circumference ≥102 cm in men and ≥88 cm in women) [26]. ^f^ Metabolically healthy: less than two risk factors of the metabolic syndrome, except waist circumference above 102 cm and 88 cm for men and women, respectively. ^g^ Metabolically unhealthy: two or more risk factors of the metabolic syndrome. Waist circumference above 102 cm and 88 cm was allowed for men and women, respectively [27].

**Table 3:** Changes in obesity and blood pressure parameters of participants completing the 6 month intervention phase with the adapted Diabetes Prevention Program (*n* = 133).

Variable	Baseline Mean ± SD	6 Months Mean ± SD	Difference to 6 Months Mean ± SD	*p*^a^ Value	*p*^b^ Value
BMI (kg/m^2^)					<0.001
Clinic 1	34.7 ± 5.20	31.8 ± 5.34	−2.97 ± 2.65 ^2,3,5^	<0.0001	
Clinic 2	34.6 ± 3.98	33.4 ± 4.26	−1.28 ± 1.51 ^1^	<0.001	
Clinic 3	34.2 ± 6.12	33.1 ± 6.11	−1.07 ± 1.87 ^1^	0.015	
Clinic 4	35.4 ± 6.54	33.5 ± 6.02	−1.90 ± 1.89	<0.0001	
Clinic 5	33.2 ± 5.17	32.0 ± 4.96	−1.26 ± 1.55 ^1^	<0.001	
Waist circumference (cm)					0.023
Clinic 1	107 ± 13.0	98.0 ± 13.3	−9.44 ± 6.86 ^3^	<0.0001	
Clinic 2	109 ± 10.6	103 ± 9.40	−5.81 ± 5.86	<0.0001	
Clinic 3	105 ± 12.4	101 ± 11.7	−3.76 ± 5.91 ^1^	0.009	
Clinic 4	110 ± 16.5	103 ± 15.9	−7.44 ± 5.59	<0.0001	
Clinic 5	103 ± 10.5	96.6 ± 9.40	−6.81 ± 6.90	<0.0001	
Body fat percentage ^c^					0.006
Clinic 1	44.5 ± 5.41	40.5 ± 7.62	−4.03 ± 4.23 ^3,4,5^	<0.0001	
Clinic 2	44.5 ± 5.84	42.5 ± 6.30	−2.00 ± 2.15	<0.001	
Clinic 3	45.1 ± 5.57	43.8 ± 5.56	−1.40 ± 2.35 ^1^	0.015	
Clinic 4	44.8 ± 5.32	43.1 ± 4.79	−1.69 ± 2.18 ^1^	<0.0001	
Clinic 5	45.3 ± 6.24	43.6 ± 5.99	−1.75 ± 2.31 ^1^	<0.001	
Systolic blood pressure (mmHg)					0.509
Clinic 1	122 ± 12.2	120 ± 12.2	−2.83 ± 13.2	0.213	
Clinic 2	119 ± 11.8	113 ± 12.4	−6.04 ± 8.96	<0.01	
Clinic 3	130 ± 18.3	123 ± 16.0	−6.67 ± 10.9	0.011	
Clinic 4	125 ± 16.6	117 ± 13.7	−8.04 ± 15.7	0.014	
Clinic 5	119 ± 11.1	116 ± 14.5	−3.19 ± 12.3	0.190	
Diastolic blood pressure (mmHg)					0.739
Clinic 1	77.3 ± 6.81	72.8 ± 9.30	−4.54 ± 9.14	0.006	
Clinic 2	74.8 ± 9.98	70.2 ± 10.2	−4.60 ± 9.29	0.021	
Clinic 3	78.0 ± 12.2	75.0 ± 7.59	−3.10 ± 8.61	0.115	
Clinic 4	75.8 ± 11.7	73.2 ± 9.27	−2.56 ± 8.52	0.146	
Clinic 5	75.7 ± 7.75	71.9 ± 9.83	−3.81 ± 8.95	0.036	

^1^ Clinic 1 (*n* = 35), ^2^ clinic 2 (*n* = 25), ^3^ clinic 3 (*n* = 21), ^4^ clinic 4 (*n* = 25), and ^5^ Clinic 5 (*n* = 27). *p*^a^ value by comparing the basal value and the final value with a paired *t*-test. *p*^b^ value of the comparison between the clinics at 6 months using a one-way ANOVA test. The superscript numbers indicate the clinics in which there are significant differences, *p* < 0.05 with a Bonferroni test. ^c^ Body fat percentage (*n* = 131).

## Supplementary Materials

The following are available online at https://www.mdpi.com/2072-6643/11/10/2324/s1, Table S1: Baseline characteristics of participants completing and not completing (drop-outs) the 6 months of intervention; Supplementary Material: Outcomes at 12 months of lifestyle intervention.
