# Supplementary material for: Effectiveness of the Diabetes Prevention Program for Obesity Treatment in Real World Clinical Practice in a Middle-Income Country in Latin America
Source: Nutrients. 2019 Oct 1;11(10):2324. doi: 10.3390/nu11102324 (PMC6835923; doi:10.3390/nu11102324)
Supplement: Supplementary file 1 [file nutrients-11-02324-s001.zip › supplementary/Supplementary material.docx]

**Supplementary material 1.** Outcomes at 12-month of lifestyle intervention.

Reasons why participants reported leaving the study at 12 months

Some of the reasons why participants reported leaving the study at this stage were: time impossibility to attend, change of residence, among others. There was a change of nutrition interns during the study, who usually have a 12-month commitment, which also affected participant retention. In addition, 4 exclusion criteria were reported: 3 pregnancies and 1 alternative treatment for weight loss

Results in other outcome variables at 12 months

The completers' analysis showed body weight was significantly reduced in all clinics; in clinic 1 it was -9.30±9.44 kg (*p*<0.0001), -3.23±4.18 kg (*p*<0.01) in clinic 2, -3.39±5.40 kg (*p*=0.020) in clinic 3, -4.46±5.15 kg (*p*=0.001) in clinic 4 and -3.30±5.66 kg (*p*=0.013) in clinic 5. The intention to treat analysis for this primary variable showed the same findings in body weight with smaller magnitude. Body weight was reduced in clinic 1 (4.15±7.72 kg, *p*<0.001), in clinic 2 (1.03±2.98 kg, *p*=0.02), in clinic 3 (1.84±4.61 kg, *p*=0.01), clinic 4 (1.77±3.83) kg, *p*=0.002) and in clinic 5 (1.40±3.98 kg, *p*=0.015), with no significant differences between clinics (*p*>0.05).

There was also a significant effect on BMI and waist circumference but not on body fat percentage or blood pressure (*p*>0.05). The completers' analysis showed that BMI decreased significantly (*p*<0.05) in each of the clinics from -3.52±3.63 to -1.23±1.57 kg/m^2^. Waist circumference decrease varied from -10.1±7.86 to -6.05±5.28 cm (*p*<0.01), while body fat percentage was significantly reduced (*p*<0.05) in three clinics, with variations of -4.84±5.93 to -1.11±1.80%. The decrease in systolic blood pressure was not significant in any clinic, while the diastolic blood pressure behaved similarly, with only a significant decrease observed in clinic 1 (7.27±15.6, *p*=0.04). The 12-month evaluation also included measurement of biochemical parameters, but only 54 of the 95 participants that completed this 12-month period had their blood collected for assessment (representing 23% of those who started the intervention). Due to the small amount of data in these secondary variables, this information is not shown.

Adverse effects

Since this type of intervention is considered very low risk, there was no formal record taken of adverse events.
