# Supplementary material for: Effectiveness of the Diabetes Prevention Program for Obesity Treatment in Real World Clinical Practice in a Middle-Income Country in Latin America
Source: Nutrients. 2019 Oct 1;11(10):2324. doi: 10.3390/nu11102324 (PMC6835923; doi:10.3390/nu11102324)
Supplement: Supplementary file 1 [file nutrients-11-02324-s001.zip › supplementary/Table S1.docx]

Supplementary material.

Table 1. Baseline characteristics of participants completing and not completing (drop-outs) the six-months of intervention.

| **Variable** | **Clinic 1** | | | **Clinic 2** | | | **Clinic 3** | | | **Clinic 4** | | | **Clinic 5** | | |
| --- | --- | --- | --- | --- | --- | --- | --- | --- | --- | --- | --- | --- | --- | --- | --- |
|  | **NC** | **C** | ***P* Value^a^** | **NC** | **C** | ***P* Value^a^** | **NC** | **C** | ***P* Value^a^** | **NC** | **C** | ***P* Value^a^** | **NC** | **C** | ***P* Value^a^** |
| **Female, n (%)** | 11 (73.3) | 26 (74.3) | 0.944 | 17 (85.0) | 18 (72.0) | 0.297 | 18 (90.0) | 18 (85.7) | 0.675 | 20 (83.3) | 20 (80.0) | 0.763 | 16 (64.0) | 24 (88.9) | 0.033 |
| **Age, mean ± SD, y** | 40.6 ±10.8 | 43.5 ±11.7 | 0.414 | 45.2 ±8.90 | 44.8  ±9.84 | 0.902 | 44.3 ±8.04 | 48.2 ±8.05 | 0.130 | 41.9 ±11.6 | 47.2 ±8.10 | 0.069 | 41.6 ±9.59 | 46.4 ±9.94 | 0.082 |
| **Education, n (%)** |  |  | 0.821 |  |  | 0.267 |  |  | 0.427 |  |  | 0.267 |  |  | 0.504 |
| **Elementary school** | 1  (6.67) | 2 (5.71) |  | 4 (20.0) | 10 (40.0) |  | 1 (5.00) | 4  (19.1) |  | 4 (16.7) | 5 (20.0) |  | 3 (12.0) | 3 (11.1) |  |
| **High school** | 5  (33.3) | 15 (42.9) |  | 11 (55.0) | 7  (28.0) |  | 7 (35.0) | 7  (33.3) |  | 12 (50.0) | 8 (32.0) |  | 9 (36.0) | 7 (25.9) |  |
| **College/University** | 8  (53.3) | 14 (40.0) |  | 3 (15.0) | 6  (24.0) |  | 9 (45.0) | 9  (42.9) |  | 8 (33.3) | 9 (36.0) |  | 9 (36.0) | 15 (55.6) |  |
| **Postgraduate** | 1  (6.67) | 4 (11.4) |  | 2 (10.0) | 2  (8.00) |  | 3 (15.0) | 1  (4.76) |  | 0 (0.00) | 3 (12.0) |  | 4 (16.0) | 2 (7.41) |  |
| **Monthy income, n (%)^c^** |  |  | 0.061 |  |  | 0.754 |  |  | 0.251 |  |  | 0.398 |  |  | 0.056 |
| **<296 US$** | 1  (6.67) | 11 (31.4) |  | 10 (50.0) | 8  (32.0) |  | 1 (5.00) | 1  (4.76) |  | 6 (25.0) | 7 (28.0) |  | 3 (12.0) | 3 (11.1) |  |
| **296 to 592 US$** | 6  (40.0) | 4 (11.4) |  | 4 (20.0) | 6  (24.0) |  | 4 (20.0) | 6  (28.6) |  | 9 (37.5) | 9 (36.0) |  | 6 (24.0) | 11 (40.7) |  |
| **592 to 1,185 US$** | 4  (26.7) | 11 (31.4) |  | 3 (15.0) | 4  (16.0) |  | 9 (45.0) | 12 (57.1) |  | 7 (29.2) | 7 (28.0) |  | 13 (52.0) | 7 (25.9) |  |
| **1,185 to 1,777 US$** | 1  (6.67) | 6 (17.1) |  | 2 (10.0) | 4 (16.0) |  | 6 (30.0) | 1  (4.76) |  | 2 (8.3) | 0 (0.00) |  | 3 (12.0) | 1 (3.70) |  |
| **≥1, 777 US$** | 3  (20.0) | 3 (8.57) |  | 1 (5.00) | 3  (12.0) |  | 0 (0.00) | 1  (4.76) |  | 0 (0.00) | 2 (8.00) |  | 0 (0.00) | 5 (18.5) |  |
| **Marital status, n (%)** |  |  | 0.543 |  |  | 0.971 |  |  | 0.389 |  |  | 0.153 |  |  | 0.297 |
| **Single** | 6  (40.0) | 10 (28.6) |  | 3 (15.0) | 4  (16.0) |  | 5 (25.0) | 2  (9.52) |  | 8 (33.3) | 5 (20.0) |  | 5 (23.8) | 9 (33.3) |  |
| **Married** | 9  (60.0) | 21 (60.0) |  | 15 (75.0) | 19 (76.0) |  | 14 (70.0) | 17 (80.1) |  | 11 (45.8) | 16 (64.0) |  | 16 (76.2) | 16 (59.3) |  |
| **Divorced** | 0  (0.00) | 3 (8.57) |  | 2 (10.0) | 2  (8.00) |  | 1 (5.00) | 2  (9.52) |  | 2 (8.33) | 4 (16.0) |  | 0 (0.00) | 0 (0.00) |  |
| **Widowed** | 0  (0.00) | 1 (2.86) |  | 0 (0.00) | 0  (0.00) |  | 0 (0.00) | 0  (0.00) |  | 3 (12.5) | 0 (0.00) |  | 0 (0.00) | 2 (7.41) |  |
| **Diseases by self-report, n (%)** |  |  |  |  |  |  |  |  |  |  |  |  |  |  |  |
| **Type 2 diabetes** | 1  (6.67) | 2 (5.71) | 0.897 | 3 (15.0) | 5  (20.0) | 0.663 | 5 (25.0) | 5  (23.8) | 0.929 | 4 (16.7) | 4 (16.0) | 0.950 | 2 (8.00) | 4 (14.8) | 0.442 |
| **Hypertension** | 4  (26.7) | 6 (17.1) | 0.440 | 6 (30.0) | 6  (24.0) | 0.651 | 7 (35.0) | 8  (38.1) | 0.837 | 9 (37.5) | 6 (24.0) | 0.305 | 4 (16.0) | 6 (22.2) | 0.569 |
| **Abnormal lipids** | 0  (0.00) | 2 (5.71) | 0.345 | 3 (15.0) | 3  (12.0) | 0.769 | 1 (5.00) | 0  (0.00) | 0.300 | 2 (8.33) | 2 (8.00) | 0.966 | 1 (4.00) | 1  (3.70) | 0.956 |
| **Hypothyroidism** | 1  (6.67) | 4 (11.4) | 0.607 | 2 (10.0) | 1  (4.00) | 0.423 | 5 (25.0) | 7  (33.3) | 0.558 | 3 (12.5) | 1 (4.00) | 0.277 | 3 (12.0) | 5 (18.5) | 0.515 |
| **Depression** | 0  (0.00) | 1 (2.86) | 0.508 | 1 (5.00) | 2  (8.00) | 0.688 | 2 (10.0) | 2  (9.52) | 0.959 | 1 (4.17) | 1 (4.00) | 0.976 | 0 (0.0) | 0 (0.00) | --- |
| **Height, mean ± SD, m** | 1.63  ±0.11 | 1.63 ±0.08 | 0.831 | 1.66 ±0.07 | 1.63  ±0.07 | 0.133 | 1.59 ±0.06 | 1.60 ±0.07 | 0.875 | 1.63 ±0.09 | 1.63  ±0.07 | 0.924 | 1.65 ±0.11 | 1.61 ±0.07 | 0.092 |
| **Weight, mean ± SD, kg** | 92.2 ±15.9 | 92.6 ±19.6 | 0.951 | 97.4 ±14.6 | 91.7  ±11.8 | 0.155 | 88.9 ±14.1 | 86.8 ±13.2 | 0.620 | 92.5 ±15.1 | 94.7  ±21.2 | 0.670 | 101  ±22.3 | 85.8 ±13.0 | 0.003 |
| **BMI, mean ± SD, kg/m^2^** | 34.6 ±5.41 | 34.7 ±5.20 | 0.930 | 35.4 ±5.31 | 34.6  ±3.98 | 0.567 | 35.0 ±5.40 | 34.2 ±6.11 | 0.656 | 34.8 ±5.00 | 35.4  ±6.54 | 0.745 | 36.9 ±5.73 | 33.2 ±5.17 | 0.020 |
| **Waist circumference, mean ± SD, cm** | 109  ±9.50 | 107  ±13.0 | 0.777 | 109 ±12.7 | 109  ±10.6 | 0.868 | 108  ±11.7 | 105  ±12.4 | 0.495 | 108 ±10.6 | 110  ±16.5 | 0.609 | 113 ±16.7 | 103 ±10.5 | 0.017 |
| **Body fat percentage, mean ± SD^d^** | 44.3 ±7.46 | 44.6 ±5.42 | 0.841 | 45.4 ±4.55 | 44.7  ±5.78 | 0.669 | 46.6 ±4.80 | 45.2 ±5.57 | 0.400 | 44.4 ±5.93 | 44.8  ±5.32 | 0.793 | 44.4 ±6.24 | 45.3 ±6.24 | 0.593 |
| **Systolic blood pressure, mean ± SD, mmHg** | 120  ±16.3 | 122  ±12.2 | 0.497 | 125 ±12.1 | 119  ±11.8 | 0.094 | 120  ±12.0 | 130  ±18.3 | 0.044 | 121 ±15.3 | 125  ±16.6 | 0.416 | 125 ±11.0 | 119 ±11.1 | 0.079 |
| **Diastolic blood pressure, mean ± SD, mmHg** | 75.5 ±11.6 | 77.3 ±6.81 | 0.493 | 80.0 ±6.13 | 74.8  ±9.98 | 0.049 | 76.6 ±8.93 | 78.0 ±12.2 | 0.657 | 75.4 ±10.8 | 75.8  ±11.7 | 0.906 | 78.6 ±12.8 | 75.7 ±7.75 | 0.331 |
| **Fasting glucose, mean ± SD, mg/dL** | 88.9 ±20.1 | 86.3 ±32.2 | 0.768 | 96.2  ±27.9 | 97.9 ±42.6 | 0.876 | 83.1 ±15.6 | 86.4 ±15.4 | 0.496 | 84.3 ±17.0 | 91.2  ±24.9 | 0.267 | 85.3 ±19.6 | 84.7 ±26.2 | 0.928 |
| **Fasting insulin, median (P25, P75), µU/mL^e^** | 7.31  (4.48, 13.0) | 6.63 (5.11, 11.8) | 0.898^b^ | 6.27 (3.87, 10.8) | 6.06 (4.39, 11.9) | 0.891^b^ | 5.90 (4.55, 8.09) | 6.07  (4.49, 8.60) | 0.746^b^ | 5.98 (4.16, 7.24) | 5.27  (3.24, 8.17) | 0.391^b^ | 5.83  (3.14, 10.8) | 4.93  (4.06, 8.46) | 0.904^b^ |
| **HOMA-IR, median (P25, P75)^f^** | 1.41  (0.88, 2.96) | 1.31 (1.01, 2.39) | 0.990 ^b^ | 1.45 (0.80, 2.54) | 1.21  (0.81, 2.97) | 0.922^b^ | 1.16  (0.91, 1.74) | 1.15  (0.86, 2.37) | 0.694^b^ | 1.21 (0.97, 1.49) | 1.06  (0.60, 1.86) | 0.733^b^ | 1.09  (0.59, 2.21) | 0.95  (0.71, 1.91) | 0.872^b^ |
| **Triglycerides, mean ± SD, mg/dL** | 136  ±62.1 | 138  ±81.9 | 0.939 | 185 ±87.4 | 161  ±71.6 | 0.318 | 132  ±121 | 177  ±156 | 0.300 | 134 ±63.2 | 164  ±68.4 | 0.126 | 150 ±68.4 | 135 ±74.6 | 0.453 |
| **Total Cholesterol, mean ± SD, mg/dL** | 174  ±48.8 | 169  ±27.5 | 0.637 | 199 ±37.2 | 194  ±50.2 | 0.699 | 176  ±54.1 | 181  ±57.4 | 0.761 | 176 ±35.2 | 197  ±34.0 | 0.041 | 191 ±39.4 | 190 ±46.9 | 0.920 |
| **HDL Cholesterol, mean ± SD, mg/dL** | 51.7 ±15.9 | 53.0  ±10.5 | 0.728 | 47.3 ±12.7 | 50.6  ±17.4 | 0.477 | 49.3 ±11.9 | 42.2 ±12.9 | 0.076 | 46.2 ±12.9 | 45.9  ±11.3 | 0.934 | 37.7 ±10.6 | 43.6 ±11.8 | 0.063 |
| **LDL Cholesterol, mean ± SD, mg/dL** | 95.5  ±44.5 | 89.3  ±28.1 | 0.555 | 115 ±36.1 | 111  ±44.3 | 0.754 | 100  ±44.2 | 103  ±36.5 | 0.834 | 103 ±33.1 | 119  ±31.5 | 0.105 | 123 ±44.5 | 119 ±45.7 | 0.756 |
| **Aspartate aminotransferase, mean ± SD, U/L** | 13.4 ±5.51 | 14.9 ±5.78 | 0.385 | 15.3 ±4.42 | 16.3  ±3.39 | 0.354 | 17.9 ±7.30 | 18.9 ±7.30 | 0.656 | 16.1 ±5.66 | 18.2  ±8.57 | 0.307 | 19.9 ±7.74 | 20.6 ±9.99 | 0.778 |
| **Alanine aminotransferase, mean ± SD, U/L** | 13.7  ±4.26 | 14.7 ±4.20 | 0.428 | 15.5 ±4.17 | 17.1  ±4.54 | 0.234 | 15.8 ±5.79 | 15.6 ±4.64 | 0.924 | 13.8 ±4.19 | 17.8  ±6.54 | 0.014 | 19.7 ±6.71 | 19.9 ±7.58 | 0.913 |
| **Metabolic síndrome, n (%)^g^** | 2  (13.3) | 12 (34.3) | 0.131 | 15 (75.0) | 15 (60.0) | 0.289 | 10 (50.0) | 13 (61.9) | 0.443 | 12 (50.0) | 15 (60.0) | 0.482 | 15 (60.0) | 10 (37.0) | 0.098 |
| **Metabolically healthy, n (%)^h^** | 13 (86.7) | 23  (65.7) | 0.131 | 5 (25.0) | 10 (40.0) | 0.289 | 10 (50.0) | 8  (38.1) | 0.443 | 12 (50.0) | 10 (40.0) | 0.482 | 9 (36.0) | 16  (59.3) | 0.093 |
| **Metabolically unhealthy, n (%)^i^** | 2  (13.3) | 12  (34.3) |  | 14 (70.0) | 15 (60.0) |  | 10 (50.0) | 13 (61.9) |  | 12 (50.0) | 15 (60.0) |  | 16 (64.0) | 11  (40.7) |  |

NC: Non-Completers, C: Completers ; ^a^*P* value by comparing the basal value of the non-completers vs. completers by independent sample t test for variables with normal distribution or ^b^Mann-Whitney U-test for variables with non-normal distribution. ^c^Exchange rate: 16.88 Mexican pesos per US dollar as of September, 2015. ^d^Percentage of fat: n= 235, ^e^fasting insulin: n=226, and ^f^HOMA-IR (Homeostatic Model Assessment for Insulin Resistance): n= 220. Conventional unit conversion factors: To convert mg/dL glucose to mmol/L, multiply mg/dL by 0.0555; to convert mg/dL triglyceride to mmol/L, multiply mg/dL by 0.0113. To convert mg/dL total cholesterol, LDL-C, and HDL-C to mmol/L, multiply mg/dL by 0.026. ^g^Metabolic syndrome: Definition according to the National Expert Panel on Detection, Evaluation, and Treatment of High Blood Cholesterol in Adults (NCEP-ATP-III) update from 2005. Three or more of the following risk factors — blood pressure (systolic/diastolic ≥130/85 mm Hg), triglycerides (≥150 mg/dL), HDL-cholesterol (<40 mg/dL in men and <50 mg/dL in women), fasting glucose (≥100 mg/dL) or taking medicine for the mentioned risk factors, abdominal obesity (waist circumference ≥102 cm in men and ≥88 cm in women) [25]. ^h^Metabolically healthy: Less than two risk factors of the metabolic syndrome except waist circumference above 102 cm and 88 cm for men and women respectively. ^i^Metabolically unhealthy: Two or more risk factors of the metabolic syndrome. Waist circumference above 102 cm and 88 cm was allowed for men and women respectively [26].
